# Microfluidics-Based Approaches to the Isolation of African Trypanosomes

**DOI:** 10.3390/pathogens6040047

**Published:** 2017-10-05

**Authors:** Michael P. Barrett, Jonathan M. Cooper, Clément Regnault, Stefan H. Holm, Jason P. Beech, Jonas O. Tegenfeldt, Axel Hochstetter

**Affiliations:** 1 Wellcome Centre for Molecular Parasitology, Institute of Infection, Immunity and Inflammation, College of Medical, Veterinary and Life Sciences, University of Glasgow, Glasgow G12 8TA, UK; Michael.Barrett@glasgow.ac.uk (M.P.B.); c.regnault.1@research.gla.ac.uk (C.R.); 2 Division of Biomedical Engineering, School of Engineering, University of Glasgow, Glasgow G12 8LT, UK; Jon.Cooper@glasgow.ac.uk; 3 Division of Solid State Physics and Nano Lund, Lund University, PO Box 118, S-221 00 Lund, Sweden; stefan.holm@ftf.lth.se (S.H.H.); jason.beech@ftf.lth.se (J.P.B.); jonas.tegenfeldt@ftf.lth.se (J.O.T.)

**Keywords:** trypanosomes, microfluidics, separation, isolation, DLD, dielectrophoresis, drug testing, concentration ramping, optical trap, diagnosis

## Abstract

African trypanosomes are responsible for significant levels of disease in both humans and animals. The protozoan parasites are free-living flagellates, usually transmitted by arthropod vectors, including the tsetse fly. In the mammalian host they live in the bloodstream and, in the case of human-infectious species, later invade the central nervous system. Diagnosis of the disease requires the positive identification of parasites in the bloodstream. This can be particularly challenging where parasite numbers are low, as is often the case in peripheral blood. Enriching parasites from body fluids is an important part of the diagnostic pathway. As more is learned about the physicochemical properties of trypanosomes, this information can be exploited through use of different microfluidic-based approaches to isolate the parasites from blood or other fluids. Here, we discuss recent advances in the use of microfluidics to separate trypanosomes from blood and to isolate single trypanosomes for analyses including drug screening.

## 1. Introduction

African trypanosomes are protozoan parasites that cause disease in livestock (including *Trypanosoma congolense*, *T. vivax*, *T. evansi*, *T. brucei brucei*) and humans (*T. b. rhodesiense* and *T. b. gambiense*). The parasites are transmitted by various routes. Tsetse flies of the *Glossina* genus transmit many key species, but other biting flies can transmit *T. evansi* and *T. vivax*. In the case of *T. equiperdum*, the disease is transmitted venereally in horses.

**Figure 1:**
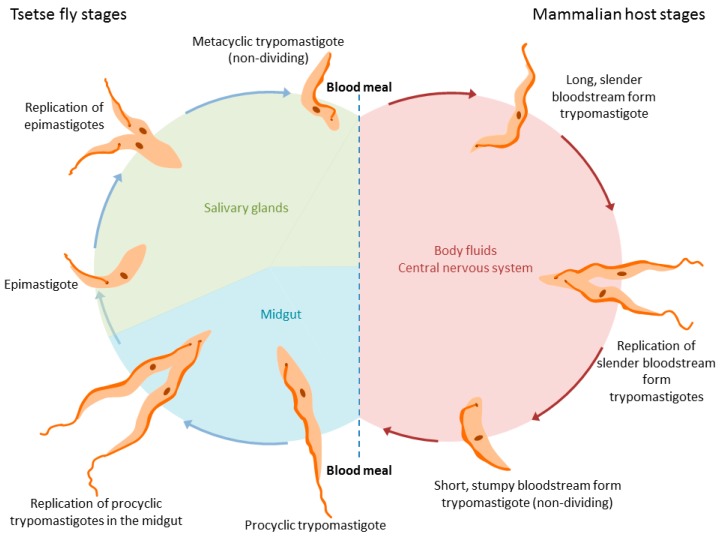
Life cycle of *Trypanosoma brucei* spp. in the tsetse fly (**left**) and the mammalian hosts (**right**).

The life cycle of trypanosomes is, in general, complex and depends on the species. In the best-studied *T. brucei* group, two key forms are generally considered in the mammalian hosts (proliferating long slender forms and non-proliferating short stumpy forms, pre-adapted for transmission to the tsetse fly; see Figure 1), and multiple forms within the vector (including the midgut proliferative procyclic trypomastigote form and the metacyclic trypomastigote form found in salivary glands pre-adapted for transfer back to mammals) [1]. During infection, trypanosomes enter the host’s bloodstream or lymphatic system during a blood meal of the tsetse fly. Later, trypanosomes also enter the central nervous system, where they induce neurological disorders and dysregulate the sleep–wake cycle of their host [2]. The exact route from blood to the brain is still not completely elucidated: the current model, in which trypanosomes cross directly from the blood to the brain via the blood–brain barrier, has recently been challenged. In the proposed model, trypanosomes cross from the blood into the cerebrospinal fluid (CSF) and possibly via the Virchow–Robin space into the brain, circumventing the blood–brain barrier entirely [2]. In a recent review, Mogk et al. discuss both models and support the latter one, hypothesizing the existence of a chronic infection stage in the meninges [2]. Recently, it has been shown that trypanosomes can be found in skin [3,4] and fatty tissues [5,6]; indeed, evidence indicates these parasites can reside and proliferate in a wide range of organs including the heart (which may account for significant morbidities and mortalities [7,8]) as well as the genital organs [9].

In spite of the flagellum offering little motility in the bloodstream, where the force of blood flow exceeds that produced by the flagellum, it is likely to play a crucial role in motility within other environments in which the trypanosomes find themselves, for example, in other organs where it does generate force required for motility. It also plays a key role in evading the immune system [10]. Employing optical tweezers, Stellamanns et al. measured the force generated by the trypanosome flagellar motor to be about 1 pN, while the power output amassed to $5.9\times 10^{-16}\;W$, about nine times more than needed for its propulsion alone in a static fluid environment [11]. The excess energy of each stroke of the flagellum (about $4.0\times 10^{-17}\;J$) generates a hydrodynamic drag force, which draws any surface-bound antibodies towards the flagellar pocket, a region through which the flagellum leaves the cell and where the sub-pellicular microtubule array is absent, creating a specialised space for endocytosis and exocytosis. Antibodies swept to the flagellar pocket are endocytosed and subsequently digested within the phagolysosomal system [10]. Together with their ability to express, in semi-sequential fashion, a single variant surface glycoprotein gene, from a repertoire of many hundreds [12], the parasites cans sustain infections for many months or even years.

Diagnosis of trypanosomiasis still depends upon identification of trypanosomes in blood, a medium in which there may be up to 10^9^ trypanosomes per mL, compared with only 10^7^ per mL in culture medium [13,14]. In order to obtain high numbers of trypanosomes for visual identification or for molecular and biochemical analysis, in blood, a process of enrichment may be required. Physicochemical properties have long been exploited to enrich for trypanosomes from whole blood. For instance, the microhematocrit centrifugation technique (mHCT) is based on the difference in density between trypanosomes and red blood cells. After centrifugation of whole blood at high speed in anticoagulant-coated capillary tubes, trypanosomes can be collected in the buffy coat, that is, the leukocyte-containing layer that sits at the interface between the erythrocyte pellet and plasma [15]. Furthermore, even purer populations of trypanosomes can be obtained using the mini-anion-exchange centrifugation technique (mAECT). This method is based on the fact that the exposed surface charge of these parasites is neutral due to their being sheathed by the contiguous variant antigen coat that protects their surface from immunoeffector molecules. Blood cells, by contrast, are negatively charged in neutrally buffered solutions due to the exposed phospholipid bilayer. Anion-exchange substrates, such as a diethylaminoethyl cellulose (DEAE-C) matrix, therefore bind red blood cells and leukocytes while trypanosomes remain unbound and pass freely through anion-exchange columns [16,17]. The mAECT was adapted to be performed on the buffy coat, after centrifugation of 5 mL of whole blood (mAECT–BC), which was shown to significantly improve the sensitivity of this diagnostic test [18]. A recent comparative study reported that mAECT–BC is the parasitological technique for blood examination that offers the best performance in the context of gambiense human African trypanosomiasis (HAT) diagnosis [19].

In this review, we discuss recent advances in the use of microfluidic-based approaches to separate trypanosomes form blood using a range of different physicochemical parameters.

## 2. Separation by Dielectrophoresis

**Figure 2:**
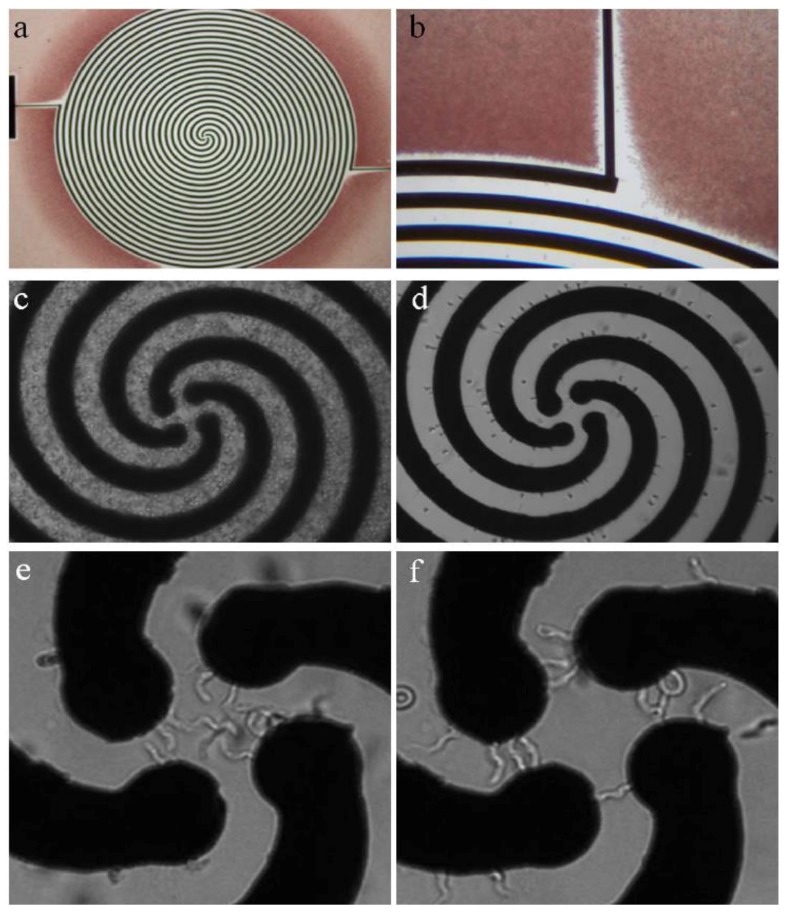
Dielectrophoretic separation device for trypanosomes in blood matrix. (**a**,**b**) The spiral array has a diameter of 2.9 mm, electrode width and spacing equals 0.03 mm. During the separation, red blood cells (RBCs) are pushed away from the electrode (red). (**c**) Micrograph of artificially infected blood. (**d**) Murine RBCs are forced towards the outer edges of the electrode array. (**e**) Trypanosomes amass in the centre of the device, moving around in circles when exposed to AC (**f**) or being trapped between neighbouring electrodes of two-phasic DC. Reprinted from [20].

Trypanosomes carry no net surface electric charge—which, as stated above, underpins the ability to separate them from blood cells using anion-exchange chromatography [20]. However, in a non-uniform electric field, a dipole moment can be induced within the trypanosome that can result in a dielectrophoretic force (DEP)—resulting in movement caused by the interaction of the induced dipole moment with an electric-field gradient. Menachery et al. employed this DEP technique to enrich trypanosomes in murine blood in a four-armed spiral electrode array (see Figure 2). By applying a quadrature-phase voltage of 2 V at 140 kHz inside this spiral electrode array, they generated a traveling-wave electric field. This field separated trypanosomes from red blood cells due to their induced dipole moment, shape and size. Affected cells underwent levitation and translational motion. Red blood cells (RBCs) were pushed upwards and outwards, while trypanosomes were pulled downwards and towards the centre of the device. A key aspect of the DEP separation relates to the lateral movement of the cells depending not only on the cells’ shapes and sizes, but also on their internal polarizability, regardless of surface charge, which distinguishes the approach from existing methods used to enrich trypanosomes (e.g., centrifugation and mini-anion-exchange chromatography).

In order to optimize the separation of trypanosomes from the blood-sample matrix, it was necessary to analyze the cross-over frequencies of both cell types. The cross-over frequency is characteristic for each cell and describes the AC-frequency at which viable cells experience the reversal of DEP force direction (i.e., from force away from high electric-field density to force attracting to high electric-field density). In this case, the cross-over frequency for trypanosomes was below 140 kHz, and for RBCs above 140 kHz, thus allowing for effective separation using DEP. This difference in cross-over frequency is due primarily to differences in cell shape and size [20].

After 10 min of separation, the trypanosomes moved under the influence of the travelling wave electric field to the centre of the spiral electrode array. The voltage had to be kept at a maximum of 2 V to avoid lysis of trypanosomes at the centre of the device [20]. The limit of detection for a single spiral electrode (2.9 mm diameter) device was determined to be $1.2\times 10^{5}$ trypanosomes per mL in whole blood, which could be increased by about two orders of magnitude when using ten spiral arms with a diameter of 10 mm to process one mL of infected blood. Although this limit of sensitivity exceeds the threshold of many trypanosome infections (which can be as low as <10 parasites per mL of blood), the fact that the array was designed for a handheld device, powered by batteries, might render it useful for field work in endemic areas with scarce resources. Moreover, as the technology is refined and possibly integrated with orthogonal methods for parasite enrichment, the potential for this technique is exciting. Along with the tunability of the cross-over frequency, various pathogens, including the African trypanosomes of veterinary importance, *Trypanosoma cruzi*, *Leishmania* species, flukes or microfilarial worms could also be enriched for diagnosis with variations on the DEP theme [20].

## 3. Separation by Deterministic Lateral Displacement (DLD)

A second microfluidic approach that has been successfully applied to the separation and detection of trypanosomes employs deterministic lateral displacement (DLD), a technique that uses a structured array of obstacles inside a microfluidic channel to generate a patterned flow-field with which particles interact and, based on physical properties such as size and shape, become spatially separated.

**Figure 3:**
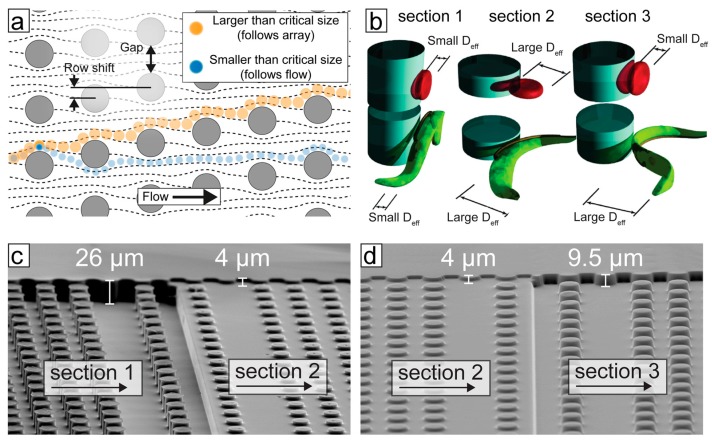
The principle of trypanosome/blood cell separation by deterministic lateral displacement (DLD). (**a**) An array of posts divides a fluid-flow into many well-defined streams. Particles smaller than the critical size follow the streams, whereas larger particles follow a trajectory defined by the geometry of the array. (**b**) The effective size of particles is a function of their shape and orientation as they flow through the device. Device depth can be used to control the orientation of blood cells and parasites to maximize differences in effective sizes. (**c**,**d**) Scanning electron microscopy images of a poly(dimethylsiloxane) (PDMS) device, designed to separate trypanosomes from blood cells; the different sections achieve different separation steps.

The basic principle of separation by DLD can be understood by considering the interaction of particles with solid obstacles in the path of a fluid flow. The centre of a particle in a laminar flow will follow a streamline unless influenced by a force perpendicular to the direction of flow; a steric interaction between particle and obstacle constitutes just such a force. In a simplified model, if a fluid streamline carrying a particle passes closer to the surface of an obstacle than the effective radius of the particle, the particle will be pushed into a neighbouring streamline. The larger the particle, the further it will be pushed. If another obstacle is placed downstream in the correct position, then all particles larger than a critical size, that were pushed far enough by the first obstacle, will again be pushed laterally with respect to the flow direction, while all particles smaller than the critical size will remain in their original streamlines. In a DLD device these small lateral displacements are repeated many times in an array of obstacles, leading to the lateral separation of particles based on their effective size at the end of the array (see Figure 3a).

The mechanism of separation by DLD was first shown by Huang et al. [21]. The strength of the technique lies in the fact that it is continuous, does not require the application of external fields other than fluid flow, is label free and separates with excellent resolution (better than 1% size difference for polystyrene spheres in the 1 µm size range). In order to design devices for specific applications, a more nuanced model than that presented above is required. Inglis [22] and Davis [23] developed an understanding of the critical size based on the array parameters shown in Figure 3a. To first approximation, the critical size is proportional to the gap size and the square root of the row shift. Studying the behaviour of erythrocytes in DLD devices, Beech et al. [24] and Henry et al. [25] developed a more detailed understanding of how the size, shape and deformability of the particles themselves contribute to their effective size and how particles could be separated by these parameters, which are highly relevant from a biological perspective. It is the difference in size and primarily in shape that is used for the separation of trypanosomes from blood using DLD.

Holm et al. [26] modified DLD devices in order to greatly improve the separation of trypanosomes from blood cells. As mentioned above, the primary requirement of a microfluidic tool to improve detection of trypanosomes is to remove (or reduce) the large background of RBCs. While, at first appearances, trypanosomes seem to be much larger than RBCs, in a standard device they are hard to separate, due to the increase in the throughput of the devices (where deep devices enable non-spherical particles to rotate such that it is their smallest dimension that defines their critical size). In shallower devices (typically shallower than the longest dimension of the particles), rotation is hindered and the effective size of the particles changes, as shown in Figure 3b. In this way, Beech et al. [24] and Holm et al. [26] showed that careful choice of device depth can maximize the difference in effective size between RBCs and trypanosomes, greatly improving separation.

In the most recent iteration, Holm et al. [27] showed a device that could deal with blood with low concentrations of parasites, with as little dilution as possible, outputting a sample stream of plasma containing parasites and close to no blood cells. The device was designed with simplicity and ease-of-use as highest priorities. In order to remove the need for expensive and power-consuming pumps, the device has one inlet only, and flow is driven using only a disposable syringe. Because of this simple, one-inlet design, all functionality must be built into the device which is able to (1) remove leukocytes in order to avoid clogging in subsequent steps, (2) create cell-free plasma and (3) transfer parasites into the cell-free plasma. The functionality in each section of the device comes from a combination of the array-spacing parameters and the depth of the channel (height of the posts). Figure 3c and d show electron-microscopy images of sections of the multi-depth device from [27].

**Figure 4:**
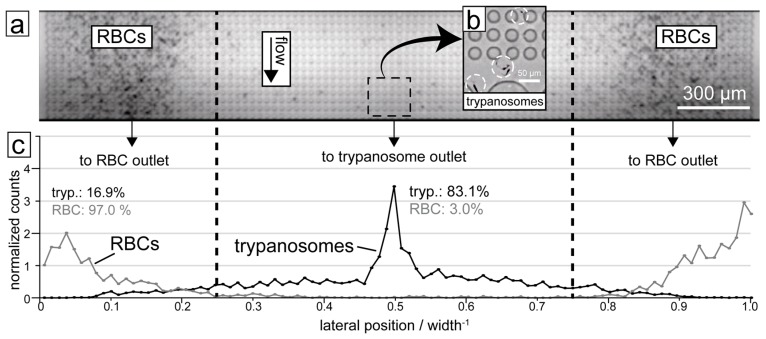
Results of trypanosome/RBC separation in the DLD device. (**a**) Micrograph showing the end section of the device with RBCs being focused to the sides. (**b**) The trypanosomes (highlighted with circles) are on the other hand focused into the centre of the device. (**c**) Quantification of a typical separation showing the distribution of the two cell types along the end section of the DLD device. 83.1% of trypanosomes are displaced into the central outlet while 97.0% of RBCs are displaced into the two side outlets. The dashed lines show the boundaries between the outlets.

Through this multi-height design, red cells, white cells and trypanosomes could all be successfully separated from each other, as shown in Figure 4.

## 4. Separation of Trypanosomes Using Optical Tweezers and Drug Screening

Optical tweezers have been used for many years to capture individual, microscopic particles through attractive forces yielded by a highly-focused laser beam. Captured particles, like solid silica spheres, hollow polymer spheres and single cells, can then be manipulated and moved as though held by remarkably fine tweezers. Optical tweezers have been used on trypanosomes for multiple purposes [11,28,29,30,31,32]. For example, optical tweezers were employed to separate and distinguish trypanosomes from other cells [30,31], to analyse their chemotactic behaviour [28] and to measure the forces created by their flagellar motors [11]. Whilst it is possible to estimate the forces and energy consumption of freely swimming cells [33], optically confined cells can be studied at higher magnification in greater detail. It is worth noting that the shape of cells and the integrated structure of their flagella have a pronounced, yet often overlooked, impact on the motility of trypanosomes [34] and cells in general, and that motility in turn is vital to cell differentiation [35,36]. Since optically confined trypanosomes are limited in their motility but retain their full mobility, a very small field of view can be used to quantify the forces and power of their flagellar motors [11]. The two distinct motility modes (tumbling and persistent/directional) have been present both in freely swimming [34,37] and optically confined trypanosomes, where directional displacement in optical confinement resulted in a persistent rotation around the point of optical confinement [11].

**Figure 5:**
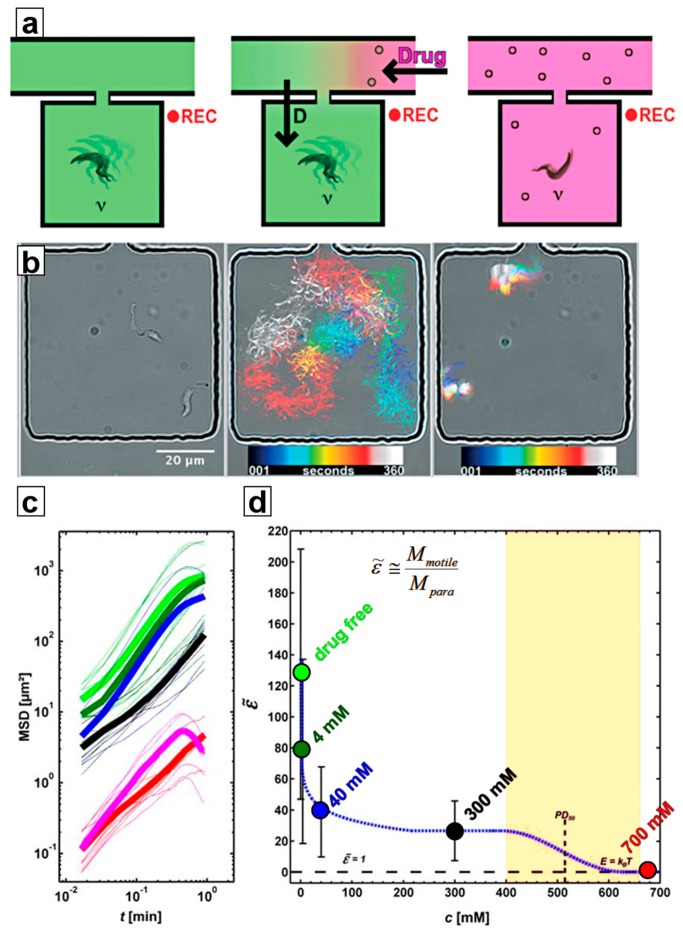
Motility recording and analysis: (**a**) Schematic of the experimental procedure (left to right): individual trypanosomes were put into microchambers, where their motility in drug-free condition was recorded. By flushing the main channel of the device, the drug concentration inside the adjacent microchambers was increased in a diffusion-controlled manner. Once the drug level had reached the desired concentration, the motility of the trypanosomes was recorded again. (**b**) (left to right) Micrograph of two trypanosomes inside a microchamber; superimposed image of the color-coded trajectory of the two trypanosomes in drug-free condition; superimposed colour-coded trajectory of two trypanosomes during drug exposure. (**c**) Mean squared displacements (MSD) of trypanosomes at various drug concentrations. (**d**) Energy output of trypanosomes (given as the motility factor $\tilde{\varepsilon }$, a multiple of the thermal energy, which causes Brownian motion) versus concentration of 2-deoxy-D-glucose). The colour code for drug concentrations in (**c**,**d**) is the same.

In addition, optical tweezers have been used to select single trypanosomes to study drug impact at the single-cell level [29]. In these studies, trypanosomes were confined individually to microfluidic compartments (microchambers) under no-flow conditions to investigate their motility patterns and to quantify their motility by calculating mean squared displacement (MSD, see Figure 5). By comparing the MSD of motile trypanosomes to the MSD of paralyzed trypanosomes, it was possible to express the energy a trypanosome consumes for propulsion in multiples of the thermal energy required to cause Brownian motion of an immobile trypanosome [29] (see Figure 5c,d).

These devices also offered a means to study the effects of drugs on trypanosomes (and other cells) using two different approaches, described in Section 4.1 and Section 4.2 below:

### 4.1. Ramping

**Figure 6:**
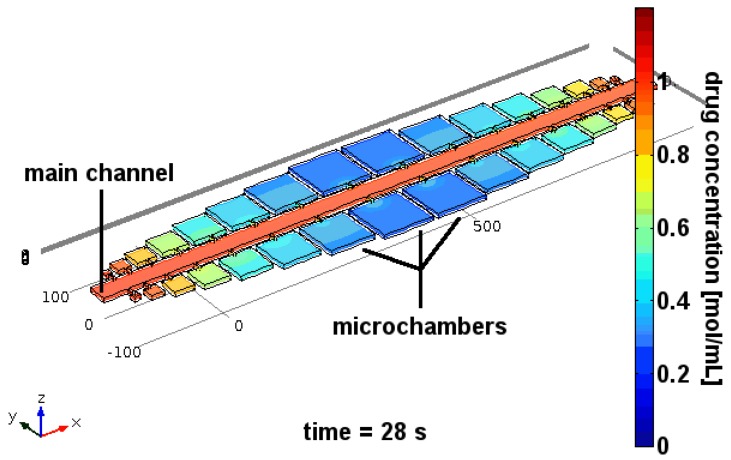
Ramping drug exposure: Cells are put into the microchambers without any drug present in the device. Then, a drug-loaded solution is pumped through the main channel at high velocity, to ensure a quasi-steady-state of maximum drug concentration in the main channel. Through the long and narrow connecting channels, drug diffuses slowly into the microchambers, ramping up the drug concentration inside the chambers in a diffusion-controlled manner. During the entire experiment, the motility of cells inside the microchambers is recorded.

In the ramping method, captured trypanosomes were sorted into larger-sized microchambers (100 × 100 µm^2^, see Figure 5b and Figure 6). The main channel that feeds the chambers is then flushed with a solution containing a high concentration of the test drug. Drug distribution into the microchambers from this reservoir is governed by diffusion rate, which ensures predictable and reproducible exposure of drug concentrations over a wide range of concentrations within minutes. Flushing of the main channel with drug-free medium then reduces the drug concentration inside the microchambers by reverse diffusion, thus stopping drug exposure at any chosen time, enabling time-dependent effects of drug exposure to be analyzed. These include differentiation between trypanostatic effects (reversible loss of motility) and trypanolysis or other irreversible effects on viability. It may even be possible to introduce methods that simulate in-vivo pharmacokinetic and pharmacodynamic (PK/PD) profiling by altering drug concentration to which parasites are exposed over time, thus mimicking in-vivo exposure where drug doses vary in time following an initial dose. The capability to distinguish between these effects offers a clear advantage over classical micotitre plate-based screening approaches.

### 4.2. Constant Exposure

The constant exposure approach offers a single-cell and observable derivative of the classical microtitre plate approaches. Microchambers loaded with cells are filled with drug solution of desired concentration. The size of the microchamber and of the connecting channel influence the time needed to establish the same drug concentration in the chamber as in the main channel (potentially as quick as a few seconds). Alternatively, cells and drug solutions can be already mixed outside the chambers after the entire device has been washed with drug solution and prior to separating trypanosomes into microchambers, where their motility can be recorded and analysed while being exposed to a constant drug level. The first approach makes possible viewing effects at the onset of drug exposure, while the second approach (premixing) ensures that all recorded data show the cells at a constant drug level, albeit after the lag time incurred by premixing outside of the microfluidic device [29].

## 5. Conclusions

Successful application of biophysical techniques in separating trypanosomes from blood cells has been of fundamental importance in diagnosing the disease caused by these parasites and enabling the molecular and biochemical analysis of separated trypanosomes. Classically, buffy-coat centrifugation and anion-exchange chromatography have been combined to enable this. New methods involving novel microfluidics approaches that can exploit the biophysical characteristics of these cells have proliferated in recent years. Here we have outlined how dielectrophoresis, deterministic lateral displacement and optical tweezer-based methods have been applied to the separation and manipulation of trypanosomes. Another approach, for example, tuning surface acoustic waves (SAW), may also find application [38] given its ability to separate particles according to their shape, size and other characteristics, including deformability. SAW was applied to create single-drop centrifugation able to separate healthy erythrocytes from malaria-infected ones [38]. In 2015, loop-mediated isothermal amplification (LAMP) was reported as a proven technique for detecting trypanosome genetic material [39,40,41,42,43,44,45,46], and combining a microfluidic device design with LAMP detection of trypanosomes offers a potential improvement in diagnostic capability. Integrating several microfluidics approaches may further improve diagnostic capability with improved speed and scale of separation. DEP, for example, could also be coupled with the shape-dependent opto-electric cell lysis [47,48,49], or specific chemical lysis [50], wherein RBCs can be selectively destroyed while preserving trypanosomes, improving their detection. The scale at which instruments exploiting these techniques can be produced is amenable to production of handheld devices with readouts readily achieved within the processing power of modern smartphones. In addition to both the human infective and veterinary animal trypanosomes, other haemoparasites may be amenable to similar approaches (e.g., *Trypanosoma cruzi*, *Leishmania* species and microfilaria). Of all these techniques, DLD and optical tweezers could potentially also be used to separate different life-cycle stages of the same pathogen (e.g., short stumpy bloodstream forms from long slender blood stream forms of *Trypanosoma brucei brucei*), based on differences in their shapes and/or sizes. Moreover, intestinal parasites such as *Giardia*, *Trichomonas* and *Entamoeba* are endowed with similar types of biophysical characteristics, and would likely be separated from stools using these approaches, too.
